# Serum Adropin Levels Are Elevated in Patients With Hyperthyroidism

**DOI:** 10.1155/2024/7144798

**Published:** 2024-10-21

**Authors:** Xin Wang, Xiaona Chang, Qiu Wang, Xiaoyu Ding, Jiaxuan Wang, Ruixiang Cui, Guang Wang, Jia Liu

**Affiliations:** ^1^Heart Center and Beijing Key Laboratory of Hypertension Research, Beijing Chao-Yang Hospital, Capital Medical University, Beijing 100020, China; ^2^Department of Endocrinology, Beijing Chao-Yang Hospital, Capital Medical University, Beijing 100020, China; ^3^Department of Endocrinology and Metabolism, Guangxi Academy of Medical Sciences and the People's Hospital of Guangxi Zhuang Autonomous Region, Nanning, Guangxi 530021, China

**Keywords:** adropin, hyperthyroidism, regression analysis, thyroid hormones

## Abstract

**Objective:** Adropin is a unique hormone, which controls metabolism and energy homeostasis. Hyperthyroidism is a disease with a high metabolic rate that affects both glucose and lipid metabolism. We aimed to investigate the change of adropin levels and the association between adropin levels and clinical parameters in patients with hyperthyroidism.

**Methods:** This cross-sectional study comprised 90 newly diagnosed patients with hyperthyroidism and 90 age- and gender-matched healthy controls. Circulating adropin levels and thyroid hormone levels were evaluated in each participant.

**Results:** Compared with the healthy controls, the hyperthyroid patients had significantly higher levels of serum adropin (*p* < 0.001). In addition, adropin levels were positively correlated with free triiodothyronine (FT3) and free thyroxine (FT4), whereas they were negatively correlated with thyroid-stimulating hormone (TSH). A multivariate linear regression analysis showed that serum adropin concentrations were independently correlated with FT3 and TSH after adjustment for age, gender, and other confounding factors (FT3: *β* = 0.231, *p* < 0.05; TSH: *β* = −0.301, *p* < 0.05).

**Conclusions:** Patients with hyperthyroidism had elevated serum adropin levels. And the serum adropin concentrations were independently correlated with the FT3 and TSH levels.

## 1. Introduction

Adropin, a highly conserved 76 amino acids peptide, is encoded by energy homeostasis-associated genes (ENHOs) [1]. Adropin is mainly expressed in the liver and brain [2, 3]. Several previous studies indicated that adropin contributed to the modulation of metabolism and energy homeostasis [4, 5]. Human studies have also confirmed a negative correlation between adropin levels and body mass index (BMI) [5]. Adropin is involved in regulating energy homeostasis by affecting lipogenic gene expression in adipose tissues while suppressing adipogenesis [2, 6]. Furthermore, adropin improves insulin sensitivity and glucose and lipid metabolism in obesity [1].

Thyroid hormones are an essential regulator of development, growth, neural differentiation, and energy homeostasis [7–9]. Thyroid hormones maintain the basal metabolic rate, modulate food intake, and influence body weight. Hyperthyroidism refers to a clinical state that results from an excessive concentration of thyroid hormones as their synthesis increase notably [10, 11]. The most common cause of hyperthyroidism is Graves' disease (GD). Recently, some rodent studies have showed that experimental hyperthyroidism leads to a significant change in adropin levels. It may be speculated that there is an association between adropin and hyperthyroidism. This study aims to investigate the change in adropin levels and the association between adropin levels and clinical parameters in patients with hyperthyroidism.

## 2. Materials and Methods

### 2.1. Study Design and Subjects

**Figure 1:**
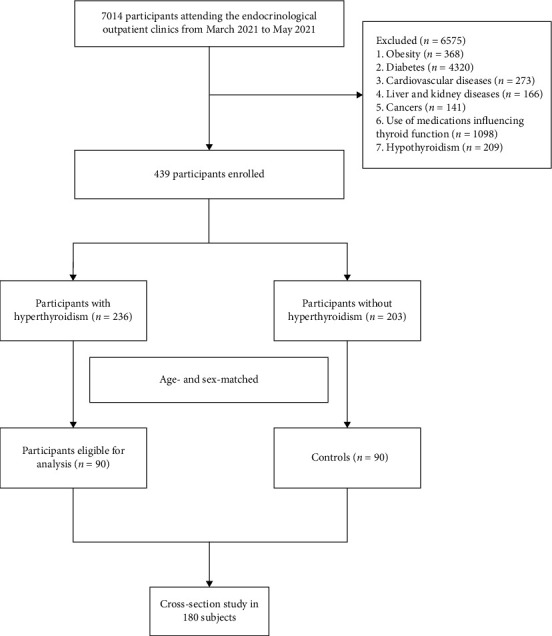
Flowchart of study procedure.

From March 2021 to May 2021, a total of 90 newly diagnosed patients with hyperthyroidism and 90 age- and sex-matched healthy participants attending the endocrinological outpatient clinics at Beijing Chao-Yang Hospital, Capital Medical University, were enrolled in this cross-sectional study. Hyperthyroidism is a clinical state characterized by increased serum FT3 and FT4 levels but decreased TSH levels (normal FT3: 2.63–5.71 pmol/L; normal FT4: 9.10–19.24 pmol/L; normal TSH: 0.350–4.940 mIU/L). The subjects with normal serum levels of FT3, FT4, and TSH; both negative antiperoxidase and antithyroglobulin antibodies; and the normal thyroid ultrasound were considered as healthy controls. The exclusion criteria include the following: those with obesity (BMI ≥ 28 kg/m^2^), diabetes, coronary disease, myocardiopathy, heart failure, chronic obstructive pulmonary disease, liver or kidney disease, autoimmune diseases, hypothyroidism, neoplastic disease (including thyroid carcinoma), history of thyroid surgery, and use of medications that influence thyroid function (Figure 1). All participants provided the informed consent in written form. Figure 1 shows the flowchart of the research procedure. The protocol of this study was approved by the Ethics Committee of Beijing Chao-Yang Hospital, Capital Medical University. All methods were carried out following relevant guidelines and regulations.

### 2.2. Clinical and Biochemical Measurements

All participants underwent routine physical examination including weight and height. Venous blood of every subject was collected in the morning after an overnight fast for biochemical, thyroid function, and adropin assays. The serum was collected by centrifugation with the speed of 3000 r/min for 10 min at 4°C and was stored at −80°C. Serum FT3, FT4, TSH, antithyroid peroxidase antibodies (TPOAb), and antithyroglobulin antibodies (TgAb) concentrations were estimated by electrochemiluminescence immunoassay using Abbott Architect i2000 (Abbott Diagnostics, Abbott Park, IL, USA). Triglyceride (TG), total cholesterol (TC), high-density lipoprotein cholesterol (HDL-C), low-density lipoprotein cholesterol (LDL-C), lipoprotein (a) (Lp(a)), and fasting blood glucose (FBG) were analyzed using a Dade Behring Dimension RXL autoanalyzer (Dade Behring Diagnostics, Marburg, Germany). Fasting insulin (FINS) was measured by the chemiluminescence immunoassay method using the Access 2 immunoassay system (Beckman Coulter, Inc., Brea, California, United States of America). Glycated hemoglobin (HbA1c) was detected with high-performance liquid chromatography using an HLC-723G7 analyzer (Tosoh Corp). Serum adropin levels were determined with enzyme-linked immunosorbent assay (ELISA) kits (Phoenix Pharmaceuticals, EK-032-35). BMI was calculated as body weight (kg)/height (m)^2^. Homeostasis model assessment of insulin resistance (HOMA-IR) was calculated as the following formula: FBG (mmol/L) × FINS (*μ*IU/mL)/22.5 [12], adipose tissue insulin resistance (Adipo-IR) was calculated as free fatty acid (FFA) (mmol/L) × FINS (*μ*IU/mL) [13] and estimated glomerular filtration rate (eGFR) was calculated as described earlier [14].

### 2.3. Statistical Analysis

All statistical analyses were performed with the 26.0 version of SPSS statistical software (SPSS Inc., Chicago, United States of America). Normally distributed data are expressed as the mean ± standard deviation (Mean ± SD), while abnormally distributed ones are expressed as the median (25th and 75th percentiles). Count data were analyzed with the chi-square test. Differences between the two groups were assessed using independent samples *t*-test for continuous variables with a normal distribution, and non-normal distribution data were analyzed using the nonparametric Mann–Whitney *U* tests. Correlation analyses were conducted using the Spearman correlation test. All tests were two-tailed, and the *p* value < 0.05 was considered statistically significant.

## 3. Results

### 3.1. Demographic Information and Clinical Characteristics of the Hyperthyroidism and Control Group

We first compared the demographic information and clinical characteristics of the hyperthyroidism and control groups. As shown in Table 1, the two groups were well matched in age and gender distributions. Regarding thyroid function, the levels of FT3 and FT4 were significantly higher, and the levels of TSH were lower in patients with hyperthyroidism than those in the control group (*p* < 0.001). As for lipid metabolism indicators, significantly lower levels of TG, HDL-C, LDL-C, and Lp(a) were observed in hyperthyroidism patients than those in control participants (*p* < 0.001). Furthermore, the levels of FBG and FINS remarkably increased in the hyperthyroidism group compared with the control group. We further compared the HOMA-IR, Adipo-IR, and eGFR between the two groups and found that all of the three parameters were notably higher in patients with hyperthyroidism in their counterparts (*p* < 0.001).

### 3.2. Correlation Analysis Between Serum Adropin Levels and Other Parameters in All Patients

We further analyzed the relationship between adropin levels and other parameters (Table 2). We found that adropin levels showed a significantly positive correlation with the FT3, FT4, FBG, HOMA-IR, Adipo-IR, and eGFR (FT3: *r* = 0.458; FT4: *r* = 0.386; FBG: *r* = 0.207; HOMA-IR: *r* = 0.192; Adipo-IR: *r* = 0.223; eGFR: *r* = 0.276; all *p* < 0.05) and a significantly negative correlation with the TSH, TC, HDL-C, and LDL-C in all patients (TSH: *r* = −0.436; TC: *r* = −0.350; HDL-C: *r* = −0.156; LDL-C: *r* = −0.285; all *p* < 0.05). No correlation was found with age, gender, BMI, TG, Lp(a), HbA1c, and FINS.

### 3.3. Multiple Linear Regression Analysis Between Adropin and Thyroid Hormones

To determine the independent effect of thyroid hormones on adropin, a multiple linear regression analysis was performed across the whole cohort (Table 3). To exclude collinearity, among all the significant variables found by Spearman's rank correlation analysis or considered clinically relevant, only sex, age, BMI, LDL-C, FBG, and eGFR were included to adjust for confounding factors. We found that FT3 and TSH levels were independently related with adropin levels (FT3: *β* = 0.231, *p* < 0.05, 95% CI = 0.003 to 0.069; TSH: *β* = −0.301, *p* < 0.05, 95% CI = −0.43 to −0.115).

## 4. Discussion

In this study, we found that serum FT3 and FT4 levels were positively correlated with adropin levels, while serum TSH levels were negatively correlated with adropin levels. Further analysis showed that the FT3 and TSH were independently related to adropin levels after adjusting for confounding factors.

We found that lipid metabolism indices were lower and glucose metabolism indices were higher in patients with hyperthyroidism than in healthy controls. Similarly, a previous research has reported that decreased levels of LDL-C, HDL-C, and TG in serum may be associated with hyperthyroidism, whereas their levels increased in hypothyroidism [15]. These results further support the important role of thyroid hormones in regulating energy homeostasis. Consistently, the total fat area, especially the visceral adipose tissue area, significantly decreased in hyperthyroidism, which may be due to increased lipid oxidation in hyperthyroidism [16]. Thyroid hormones can directly affect brown adipose tissue (BAT), and BAT can utilize glucose and lipids for thermogenesis [17–19]. Furthermore, increased glucose uptake in skeletal muscle was correlated with thyroid hormone levels [20]. We also observed a negative correlation between adropin levels and BMI, which could be attributed to a hypermetabolic state of hyperthyroidism where fat mobilization and fat-burning effect of adropin were enhanced.

It is noteworthy that serum FT3 and TSH concentrations were independently correlated with adropin concentration after adjusting for age, gender, BMI, LDL-C, FBG, and eGFR. Thyroid hormones have an important effect on metabolism, and especially the increase in these hormone levels leads to a significant increase in metabolism. It is also known that adropin, as a fat-burning hormone, plays an important role in metabolic homeostasis, control of fatty acid metabolism, and regulation of glucose tolerance [1].

In addition, obesity caused by melanocortin receptor or leptin deficiency results in reduced ENHO mRNA expression in the liver. Interestingly, caloric restriction normalized ENHO mRNA expression in the liver in melanocortin receptor–deficient mice [2]. Human studies have also demonstrated an inverse correlation between adropin levels and BMI, suggesting that low adropin is a marker of obesity [21–24]. The results from in vivo and in vitro studies show that circulating adropin levels are negatively correlated with LDL-C levels, and cholesterol suppresses ENHO mRNA expression, resulting in reduced adropin production [25]. Moreover, it was found that adropin deficiency was associated with obesity and increased insulin resistance [4]. Furthermore, obese mice treated with adropin eat less and lose body weight [2]. Moreover, adropin may promote energy homeostasis by affecting the expression of adipogenesis genes in adipose tissues while inhibiting adipogenesis [1]. Adropin may stimulate the proliferation of 3T3-L1 cells and rat primary preadipocytes and promote the function of preadipocyte and adipocyte [26].

In this research, we found a relatively insignificant association of FT4 with adropin in the regression model compared to FT3 and TSH. Physiologically, thyroid hormone regulates a wide range of genes after its activation from the prohormone, thyroxine (T4), to the active form, triiodothyronine (T3) [27–29]. In fact, FT3 is more likely to reflect hyperthyroidism. This may explain why FT3 not FT4 was more strongly correlated with adropin in the regression models.

To date, it remains unknown whether the change in adropin levels is the reason for the changes in thyroid hormones or merely a consequence of hyperthyroidism. One possible explanation is that there are TSH receptors in adipose tissues [16], and TSH affects fat metabolism, which in turn affects the adropin, a kind of adipocytokine.

It was worth noting that our study was inconsistent with the result of a previous animal study. It was found that adropin decreased significantly in the hyperthyroidism group compared to the control group [30]. The possible reason is that there are differences between animal models and human disease states. In this animal research, the rats were induced hyperthyroidism with 2-week thyroxine administration, which was not completely consistent with the clinical evolution of patients with hyperthyroidism.

There are several limitations to our study. Firstly, our research was a cross-sectional design with a relatively small sample size. Therefore, prospective studies are needed to confirm the conclusion. Secondly, there is growing evidence demonstrating that circulating adropin levels depend on diet preference [31, 32]. The differences in dietary preferences and the hypermetabolic status of patients with hyperthyroidism will affect the nutritional status of patients. Although we corrected the effect of BMI, we cannot completely eliminate the effect of the diet preferences and nutritional status in this study.

Nevertheless, the study also presents reliable hypotheses that are expected to be confirmed in future studies.

## 5. Conclusion

In summary, serum adropin levels are elevated in patients with hyperthyroidism. Serum adropin concentrations are correlated with thyroid hormone levels. Large-sampled and prospective studies are needed to further confirm this conclusion and provide a potential orientation for the prevention and treatment of hyperthyroidism.

## Figures and Tables

**Table 1:** Characteristics of hyperthyroidism patients and healthy control participants.

Variable	Control	Hyperthyroidism	*p* value
	*n* = 90	*n* = 90	
Age (years)	41.01 ± 10.61	40.37 ± 12.28	0.707
Male/female (*n*)	25/65	19/71	0.298
BMI (kg/m^2^)	22.92 ± 3.31	22.14 ± 3.49	0.130
FT3 (pmol/L)	5.04 (4.66–5.31)	22.62 (13.32–30.80)	< 0.001⁣^∗∗^
FT4 (pmol/L)	16.34 (14.93–18.18)	58.17 (36.29–81.47)	< 0.001⁣^∗∗^
TSH (mIU/L)	1.97 (1.46–2.39)	0.01 (0.01–0.01)	< 0.001⁣^∗∗^
TPOAb (U/mL)	30.45 (28.00–39.63)	836.15 (49.38–2895.65)	< 0.001⁣^∗∗^
TgAb (U/mL)	15.50 (15.00–22.63)	97.95 (27.25–245.63)	< 0.001⁣^∗∗^
TG (mmol/L)	0.97 (0.72–1.56)	1.16 (0.83–1.49)	0.193
TC (mmol/L)	5.98 ± 1.72	3.53 ± 0.58	< 0.001⁣^∗∗^
HDL-C (mmol/L)	1.50 ± 0.44	1.14 ± 0.25	< 0.001⁣^∗∗^
LDL-C (mmol/L)	3.68 ± 1.30	1.94 ± 0.62	< 0.001⁣^∗∗^
Lp(a) (mg/dL)	12.9 (7.7–26.2)	7.2 (5.1–15.7)	< 0.001⁣^∗∗^
FBG (mmol/L)	4.83 (4.56–5.18)	5.11 (4.76–5.58)	0.001⁣^∗^
HbA1C (%)	5.4 (5.2–5.5)	5.4 (5.2–5.8)	0.198
FINS (uIU/mL)	0.50 (0.39–0.71)	0.76 (0.63–0.98)	< 0.001⁣^∗∗^
HOMA-IR	1.45 (1.12–2.04)	2.75 (1.76–3.54)	< 0.001⁣^∗∗^
Adipo-IR	24.03 (16.55–42.27)	67.82 (41.68–94.29)	< 0.001⁣^∗∗^
Adropin (ng/mL)	3.10 ± 0.77	4.18 ± 1.02	< 0.001⁣^∗∗^
eGFR (mL/min/1.73 m^2^)	113.05 (104.53–121.07)	128.00 (117.33–142.50)	< 0.001⁣^∗∗^

*Note:* Normally distributed variables were expressed as mean ± standard deviation (SD), while variables with non-normal distribution were expressed as medians with the first quartile to the third quartile.

Abbreviations: Adipo-IR, adipose tissue insulin resistance; BMI, body mass index; eGFR, estimated glomerular filtration rate; FBG, fasting blood glucose; FINS, fasting insulin; FT3, free triiodothyronine; FT4, free thyroxine; HbA1C, glycated hemoglobin; HDL-C, high-density lipoprotein cholesterol; HOMA-IR, homeostasis model assessment of insulin resistance; LDL-C, low-density lipoprotein cholesterol; Lp(a), lipoprotein (a); TC, total cholesterol; TG, triglyceride; TgAb, antithyroglobulin antibodies; TPOAb, antithyroid peroxidase antibodies; TSH, thyroid-stimulating hormone.

⁣^∗^*p* < 0.05 and ⁣^∗∗^*p* < 0.001.

**Table 2:** Association between adropin and laboratory indices in all participants.

	Adropin	
	*r*	*p* value
Gender	−0.012	0.872
Age (years)	0.031	0.685
BMI (kg/m^2^)	−0.143	0.057
FT3 (pmol/L)	0.458	< 0.001⁣^∗∗^
FT4 (pmol/L)	0.386	< 0.001⁣^∗∗^
TSH (mIU/L)	−0.436	< 0.001⁣^∗∗^
TPOAb (U/mL)	0.339	< 0.001⁣^∗∗^
TgAb (U/mL)	0.261	< 0.001⁣^∗∗^
TG (mmol/L)	−0.085	0.257
TC (mmol/L)	−0.35	< 0.001⁣^∗∗^
HDL-C (mmol/L)	−0.156	0.038⁣^∗^
LDL-C (mmol/L)	−0.285	< 0.001⁣^∗∗^
Lp(a) (mg/dL)	−0.117	0.121
FBG (mmol/L)	0.207	0.006⁣^∗^
HbA1C (%)	0.097	0.266
FINS (uIU/mL)	0.16	0.054
HOMA-IR	0.192	0.022⁣^∗^
Adipo-IR	0.223	0.009⁣^∗^
eGFR (mL/min/1.73 m^2^)	0.276	< 0.001⁣^∗∗^

Abbreviations: Adipo-IR, adipose tissue insulin resistance; BMI, body mass index; eGFR, estimated glomerular filtration rate; FBG, fasting blood glucose; FINS, fasting insulin; FT3, free triiodothyronine; FT4, free thyroxine; HbA1C, glycated hemoglobin; HDL-C, high-density lipoprotein cholesterol; HOMA-IR, homeostasis model assessment of insulin resistance; LDL-C, low-density lipoprotein cholesterol; Lp(a), lipoprotein (a); TC, total cholesterol; TG, triglyceride; TgAb, antithyroglobulin antibodies; TPOAb, antithyroid peroxidase antibodies; TSH, thyroid-stimulating hormone.

⁣^∗^*p* < 0.05 and ⁣^∗∗^*p* < 0.001.

**Table 3:** Multivariate linear regression for the association of serum adropin levels with thyroid hormones.

Variables	Model 1			Model 2			Model 3		
	*β*	*p* value	95% CI	*β*	*p* value	95% CI	*β*	*p* value	95% CI
Age (years)	0.313	**0.003**⁣^∗^	(0.01, 0.047)	0.377	**< 0.001**⁣^∗∗^	(0.015, 0.053)	0.251	**0.018**⁣^∗^	(0.004, 0.041)
Gender (female1)	−0.068	0.361	(–0.514, 0.188)	−0.08	0.285	(–0.546, 0.162)	−0.061	0.404	(–0.489, 0.198)
BMI (kg/m^2^)	−0.119	0.118	(–0.081, 0.009)	−0.095	0.212	(–0.074, 0.017)	−0.124	0.094	(–0.082, 0.006)
LDL-C (mmol/L)	0.011	0.907	(–0.173, 0.194)	−0.05	0.585	(–0.231, 0.131)	0.026	0.772	(–0.149, 0.201)
FBG (mmol/L)	0.096	0.201	(–0.089, 0.419)	0.14	0.060	(–0.01, 0.492)	0.101	0.158	(–0.068, 0.414)
eGFR (mL/min/1.73 m^2^)	0.345	**0.011**⁣^∗^	(0.005, 0.037)	0.488	**< 0.001**⁣^∗∗^	(0.014, 0.045)	0.321	**0.010**⁣^∗^	(0.005, 0.034)
FT3 (pmol/L)	0.231	**0.031**⁣^∗^	(0.003, 0.069)	—	—	—	—	—	—
FT4 (pmol/L)	—	—	—	0.043	0.654	(–0.059, 0.094)	—	—	—
TSH (mIU/L)	—	—	—	—	—	—	−0.301	**0.001**⁣^∗^	(–0.43, −0.115)

*Note*: Bold values in Table 3 represent *p* < 0.05.

Abbreviations: BMI, body mass index; CI, confidence interval; eGFR, estimated glomerular filtration rate; FBG, fasting blood glucose; FT3, free triiodothyronine; FT4, free thyroxine; LDL-C, low-density lipoprotein cholesterol; TSH, thyroid-stimulating hormone.

^1^male as control.

⁣^∗^*p* < 0.05 and ⁣^∗∗^*p* < 0.001.

## Data Availability

The data used to support the findings of this study are available from the corresponding author upon request.

## References

[1] Jasaszwili M., Billert M., Strowski M. Z., Nowak K. W., Skrzypski M. (2020). Adropin as A Fat-Burning Hormone With Multiple Functions-Review of a Decade of Research. *Molecules*.

[2] Kumar K. G., Trevaskis J. L., Lam D. D. (2008). Identification of Adropin as a Secreted Factor Linking Dietary Macronutrient Intake With Energy Homeostasis and Lipid Metabolism. *Cell Metabolism*.

[3] Butler A. A., Zhang J., Price C. A. (2019). Low Plasma Adropin Concentrations Increase Risks of Weight Gain and Metabolic Dysregulation in Response to a High-Sugar Diet in Male Nonhuman Primates. *Journal of Biological Chemistry*.

[4] Ganesh Kumar K., Zhang J., Gao S. (2012). Adropin Deficiency Is Associated With Increased Adiposity and Insulin Resistance. *Obesity*.

[5] Butler A. A., Tam C. S., Stanhope K. L. (2012). Low Circulating Adropin Concentrations With Obesity and Aging Correlate With Risk Factors for Metabolic Disease and Increase After Gastric Bypass Surgery in Humans. *Journal of Clinical Endocrinology and Metabolism*.

[6] Ruiz-Ojeda F. J., Rupérez A. I., Gomez-Llorente C., Gil A., Aguilera C. M. (2016). Cell Models and Their Application for Studying Adipogenic Differentiation in Relation to Obesity: A Review. *International Journal of Molecular Sciences*.

[7] Barboza G. D. D., Guizzardi S., Talamoni N. T. D. (2015). Molecular Aspects of Intestinal Calcium Absorption. *World Journal of Gastroenterology*.

[8] Gilbert M. E., Rovet J., Chen Z., Koibuchi N. (2012). Developmental Thyroid Hormone Disruption: Prevalence, Environmental Contaminants and Neurodevelopmental Consequences. *Neurotoxicology*.

[9] Tata J. R. (2013). The Road to Nuclear Receptors of Thyroid Hormone. *Biochimica et Biophysica Acta (BBA)—General Subjects*.

[10] Kravets I. (2016). Hyperthyroidism: Diagnosis and Treatment. *American Family Physician*.

[11] Ross D. S., Burch H. B., Cooper D. S. (2016). 2016 American Thyroid Association Guidelines for Diagnosis and Management of Hyperthyroidism and Other Causes of Thyrotoxicosis. *Thyroid*.

[12] Matthews D. R., Hosker J. P., Rudenski A. S., Naylor B. A., Treacher D. F., Turner R. C. (1985). Homeostasis Model Assessment: Insulin Resistance and Beta-Cell Function From Fasting Plasma Glucose and Insulin Concentrations in Man. *Diabetologia*.

[13] Gastaldelli A., Gaggini M., DeFronzo R. A. (2017). Role of Adipose Tissue Insulin Resistance in the Natural History of Type 2 Diabetes: Results From the San Antonio Metabolism Study. *Diabetes*.

[14] Levey A. S., Stevens L. A., Schmid C. H. (2009). A New Equation to Estimate Glomerular Filtration Rate. *Annals of Internal Medicine*.

[15] Duntas L. H. (2002). Thyroid Disease and Lipids. *Thyroid*.

[16] Steinhoff K. G., Krause K., Linder N. (2021). Effects of Hyperthyroidism on Adipose Tissue Activity and Distribution in Adults. *Thyroid*.

[17] Weiner J., Hankir M., Heiker J. T., Fenske W., Krause K. (2017). Thyroid Hormones and Browning of Adipose Tissue. *Molecular and Cellular Endocrinology*.

[18] Cannon B., Nedergaard J. (2004). Brown Adipose Tissue: Function and Physiological Significance. *Physiological Reviews*.

[19] Nedergaard J., Cannon B. (2010). The Changed Metabolic World With Human Brown Adipose Tissue: Therapeutic Visions. *Cell Metabolism*.

[20] Venditti P., Reed T. T., Victor V. M., Di Meo S. (2019). Insulin Resistance and Diabetes in Hyperthyroidism: A Possible Role for Oxygen and Nitrogen Reactive Species. *Free Radical Research*.

[21] Sayın O., Tokgöz Y., Arslan N. (2014). Investigation of Adropin and Leptin Levels in Pediatric Obesity-Related Nonalcoholic Fatty Liver Disease. *Journal of Pediatric Endocrinology & Metabolism: Journal of Pediatric Endocrinology & Metabolism*.

[22] Yosaee S., Khodadost M., Esteghamati A. (2017). Metabolic Syndrome Patients Have Lower Levels of Adropin When Compared With Healthy Overweight/Obese and Lean Subjects. *American Journal of Men’s Health*.

[23] Chen R. M., Yuan X., Ouyang Q. (2019). Adropin and Glucagon-Like Peptide-2 Are Associated With Glucose Metabolism in Obese Children. *World Journal of Pediatrics*.

[24] Zang H., Jiang F., Cheng X., Xu H., Hu X. (2018). Serum Adropin Levels Are Decreased in Chinese Type 2 Diabetic Patients and Negatively Correlated With Body Mass Index. *Endocrine Journal*.

[25] Ghoshal S., Stevens J. R., Billon C. (2018). Adropin: An Endocrine Link Between the Biological Clock and Cholesterol Homeostasis. *Molecular Metabolism*.

[26] Jasaszwili M., Wojciechowicz T., Billert M., Strowski M. Z., Nowak K. W., Skrzypski M. (2019). Effects of Adropin on Proliferation and Differentiation of 3T3-L1 Cells and Rat Primary Preadipocytes. *Molecular and Cellular Endocrinology*.

[27] Mendoza A., Hollenberg A. N. (2017). New Insights Into Thyroid Hormone Action. *Pharmacology & Therapeutics*.

[28] Brent G. A. (2012). Mechanisms of Thyroid Hormone Action. *Journal of Clinical Investigation*.

[29] Cheng S. Y., Leonard J. L., Davis P. J. (2010). Molecular Aspects of Thyroid Hormone Actions. *Endocrine Reviews*.

[30] Mogulkoc R., Dasdelen D., Baltaci S. B., Baltaci A. K., Sivrikaya A. (2020). The Effect of Thyroid Dysfunction and Treatment on Adropin, Asprosin and Preptin Levels in Rats. *Hormone Molecular Biology and Clinical Investigation*.

[31] St-Onge M. P., Shechter A., Shlisky J. (2014). Fasting Plasma Adropin Concentrations Correlate With Fat Consumption in Human Females. *Obesity*.

[32] Stevens J. R., Kearney M. L., St-Onge M. P. (2016). Inverse Association Between Carbohydrate Consumption and Plasma Adropin Concentrations in Humans. *Obesity*.

